# Deep learning infers clinically relevant protein levels and drug response in breast cancer from unannotated pathology images

**DOI:** 10.1038/s41523-024-00620-y

**Published:** 2024-02-27

**Authors:** Hui Liu, Xiaodong Xie, Bin Wang

**Affiliations:** 1 College of Computer and Information Engineering, Nanjing Tech University, 211816 Nanjing, Jiangsu China; 2 Department of Cardiothoracic Surgery, The Third Affiliated Hospital of Soochow University, 213110 Changzhou, Jiangsu China

**Keywords:** Breast cancer, Translational research, Cancer imaging

## Abstract

The computational pathology has been demonstrated to effectively uncover tumor-related genomic alterations and transcriptomic patterns. Although proteomics has indeed shown great potential in the field of precision medicine, few studies have focused on the computational prediction of protein levels from pathology images. In this paper, we assume that deep learning-based pathological features imply the protein levels of tumor biomarkers that are indicative of prognosis and drug response. For this purpose, we propose wsi2rppa, a weakly supervised contrastive learning framework to infer the protein levels of tumor biomarkers from whole slide images (WSIs) in breast cancer. We first conducted contrastive learning-based pre-training on tessellated tiles to extract pathological features, which are then aggregated by attention pooling and adapted to downstream tasks. We conducted extensive evaluation experiments on the TCGA-BRCA cohort (1978 WSIs of 1093 patients with protein levels of 223 biomarkers) and the CPTAC-BRCA cohort (642 WSIs of 134 patients). The results showed that our method achieved state-of-the-art performance in tumor diagnostic tasks, and also performed well in predicting clinically relevant protein levels and drug response. To show the model interpretability, we spatially visualized the WSIs colored the tiles by their attention scores, and found that the regions with high scores were highly consistent with the tumor and necrotic regions annotated by a 10-year experienced pathologist. Moreover, spatial transcriptomic data further verified that the heatmap generated by attention scores agrees greatly with the spatial expression landscape of two typical tumor biomarker genes. In predicting the response to drug trastuzumab treatment, our method achieved a 0.79 AUC value which is much higher than the previous study reported 0.68. These findings showed the remarkable potential of computational pathology in the prediction of clinically relevant protein levels, drug response, and clinical outcomes.

## Introduction

Visual inspection of histopathology slides via an optical microscope is the routine medical examination for the clinical diagnosis of tumors. Digital pathology makes use of automated microscopy or optical magnification systems to scan and digitize traditional glass pathology slides. Computer algorithms are then employed to perform high-precision multi-field seamless stitching and processing of the images to produce whole-slide images (WSIs). In recent years, computational pathology has advanced rapidly to facilitate the automatic diagnosis of tumors. The current approach to automatic tumor diagnosis employs machine learning techniques to address the challenge of accurately identifying cancerous cells^1–4^. This approach typically involves three stages: identifying regions of interest (ROI), extracting pertinent features, and training a diagnostic model. While the accuracy of the computational methods may not be as reliable as that of experienced pathologists, they effectively reduce the workload of these medical professionals.

Deep learning has made significant strides in many fields and has also spurred advances in computational pathology. Such methods have demonstrated impressive progress in a variety of challenging clinical tasks^5–8^. For tumor diagnosis and semantic segmentation, Liu et al.^9^ proposed MSMV-PFENet to classify breast cancer histology images. Elmannai et al.^10^ combined two deep convolutional neural networks (DCNNs) to classify breast cancer as normal, benign, in situ carcinoma, and invasive carcinoma. Wang et al.^11^ developed an enhanced histology-based digital-staining algorithm based on mask-RCNN that can perform nuclear segmentation and cell classification on lung adenocarcinoma images. This algorithm can also be applied to head and neck cancer, breast cancer, and lung squamous cell cancer. Greenwald et al.^12^ developed TissueNet to segment cells and identify the boundaries of individual cells in whole-slide images. Eklund et al.^13^ created an integrated model based on Inception V3 to assist pathologists in finding, identifying, and grading prostate tumors. Wang et al.^14^ introduced a novel deep-learning model to improve breast cancer histological grading. Additionally, several studies have presented computational pathology methods for inferring genetic alterations and gene expression profiles. For example, Kather and colleagues^15^ introduced a deep learning approach for analyzing genetic alterations across various types of cancer. They successfully predicted the presence of CDC27 mutations in colorectal cancer using pathological images. Wang et al.^16^ proposed an approach called expression-morphology (EMO) to predict mRNA expression in 17,695 genes from whole-slide images and validated the spatial variability of intratumor expression through spatial transcriptomics profiling. Several recent studies have demonstrated the potential of using weakly supervised algorithms in computational pathology. For instance, our team has developed Histcode^17^, which integrated contrastive learning-based pretraining and weakly supervised learning to predict differential gene expression of cancer driver genes. Mahmood et al.^18^ developed the CLAM model, which used an attention mechanism for the subtype classification of renal cell carcinoma and non-small cell lung cancer. Schmauch et al.^19^ developed a model called HE2RNA to predict RNA-seq from pathological images and identified tumors with microsatellite instability in clinical diagnoses. Shamai et al.^20^ built a convolutional neural network (CNN)-based model to predict PD-L1 expression from H&E images. Eliceiri et al.^21^ developed the DS-MIL algorithm to classify WSIs as tumor or normal. Mahmood et al.^22^ developed SISH, an interpretable search pipeline that achieves high speed in searching histology images after training with only slide-level labels.

In recent times, the field of proteomics has demonstrated exceptional promise in the realm of precision medicine and achieved significant advances in the treatment of various forms of cancer^23–25^. For example, Gao^26^ utilized proteomics to identify two prognostic biomarkers, PYCR2 and ADH1A, for hepatocellular carcinoma. The reverse-phase protein array (RPPA)^27^, a high-throughput and highly sensitive protein microarray that employs antibodies to detect and quantify proteins and their modifications in biological samples, has shown tremendous potential in the discovery of cancer biomarkers, analysis of functional phenotypes, and elucidation of drug mechanisms. It has been assumed that protein profile changes in tumor cells cause functional changes, which can influence tumor cell morphology. So, routine histopathology tissue slides, which are ubiquitously available, can reflect such morphological changes, thereby the clinically relevant proteins could be inferred directly from digitized whole-slide images.

In this paper, we introduced a weakly supervised contrastive learning method to establish the quantitative associations between clinically relevant proteins and histopathological features in breast cancer. Specifically, whole-slide images were split into tiles and then MoCo v2 was employed to extract features at the tile-level. Attentive pooling was used to aggregate tile-level features to generate slide latent representations, which were then applied to various downstream tasks, including tumor diagnosis, quantification of protein levels, prognostic risk assessment, and prediction of response to the drug trastuzumab. Our extensive experiments showed that the proposed method achieved state-of-the-art performance in tumor diagnosis tasks, and achieved high performance in quantifying the protein levels of tumor biomarkers. To show the model interpretability, we spatially visualized the WSIs colored by attention scores of tiles and found that the regions with high scores are highly consistent with the tumor and necrotic regions annotated by an experienced pathologist. Moreover, spatial transcriptomic data further verified that the heatmaps generated by attention scores agree greatly with the spatial expression map of tumor biomarker genes. Our method achieved a 0.79 AUC value in predicting the response of breast cancer patients to the drug trastuzumab. These findings showed our method could effectively elucidate and quantify genotype–phenotype links in breast cancer.

## Results

### Multi-instance learning accurately identified tumor slides

**Fig. 1:**
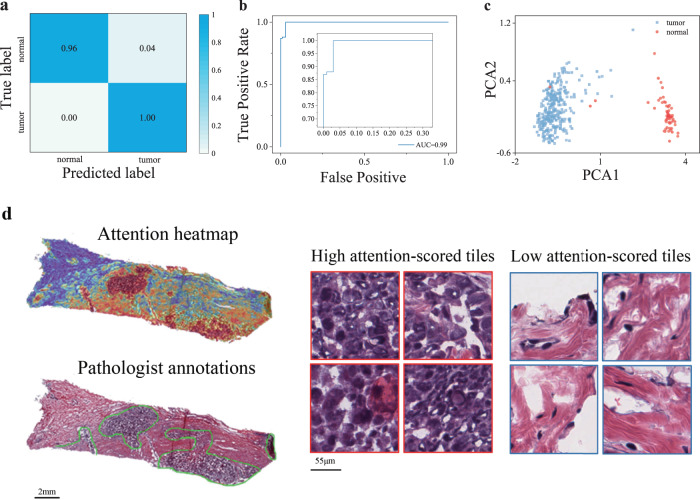
Performance evaluation on tumor diagnostic task and spatial deconvolution of tumor regions based on computational pathological feature. **a**, **b** The confusion matrix and ROC curve for tumor diagnosis task on the test set of TCGA-BRCA cohort. **c** Visualization of learned slide-level feature by PCA dimension reduction. **d** Comparison between the heatmap generated by tile-level attention scores and the tumor area annotated by a pathologist. The heatmap (upper left) is generated by the spatial deconvolution of the tiles to the original slide (lower left), coloring each tile according to its attention score. The area circled by the green line is the tumor necrosis region depicted by an experienced pathologist (lower left). The right part shows a few representative tiles with the highest and lowest attention scores.

We first tested our method for tumor diagnostic tasks on the TCGA-BRCA cohort. The pathology images (*n* = 1978, see the section “Methods”) of breast cancer were randomly split into training, validation, and independent test sets by 60%, 20%, and 20%. It is worth noting that we used only the slide-level labels to fine-tune the pre-trained model. Our method achieved 0.995 accuracy and 0.996 AUC values. As shown in Fig. 1a, b, we presented the ROC curve and confusion matrix on the independent test set. This showed that the multi-instance learning model can accurately discriminate whether WSI contains tumor cells or not. During the fine-tuned stage for tumor diagnosis, the tile-level embeddings were aggregated to build slide representation (computational histopathological features). As the network was trained to classify tumor and normal tissues, the computational histopathological features showed two clusters corresponding to each tissue class by PCA dimension reduction (Fig. 1c).

To verify the robustness of our model, we evaluated the performance of an external validation cohort obtained from the CPTAC-BRCA project. The cohort comprised 642 Whole Slide Images (WSIs) from 134 breast cancer patients. We trained our model for the tumor diagnosis task on the TCGA-BRCA cohort and then ran predictions on the CPTAC-BRCA cohort. Our model achieved an accuracy of 97.04% on such a cross-cohort validation experiment.

The slide-level classification of tumor/normal labels was made based on the attentive pooling of tile-level features, thereby the derived attention scores are highly indicative of the localization of tumor regions. To validate the spatial localization of tumor area by our model, we scaled the learned tile-level attention scores to generate heatmaps by spatial deconvolution of tiles to the original slide. For objective evaluation, an experienced pathologist was asked to annotate the tumor regions of interest (ROIs), and we visually checked whether the high-scored area was coincident with the annotated regions. As shown in Fig. 1d, the attention heatmaps showed remarkable agreement between the pathologist-annotated ROIs and our computational predictions of tumor regions. Visual inspection of tiles would reflect more human-readable pathological features. So, we showed a few tiles with high or low attention scores, which are verified by the pathologist that the low-scored tiles are mostly normal tissue, while high-scored tiles are tumor tissue.

### Effectively predicting protein levels of tumor biomarkers

The protein expression profiles of clinically relevant biomarkers produced by reverse-phase protein arrays (RPPAs) allow us to test whether the histopathological feature is predictive of protein levels of tumor biomarkers. As these proteins are enriched in diagnostic biomarkers and therapeutic targets, the establishment of the quantitative associations between computational pathology and clinically relevant proteins would greatly facilitate clinically translational applications. On the TCGA-BRCA cohort, the protein expression profile contains 223 biomarker proteins of 860 breast cancer patients. We transferred the computational pathological features obtained by contrastive learning to quantitatively predict the levels of biomarker proteins.

**Fig. 2:**
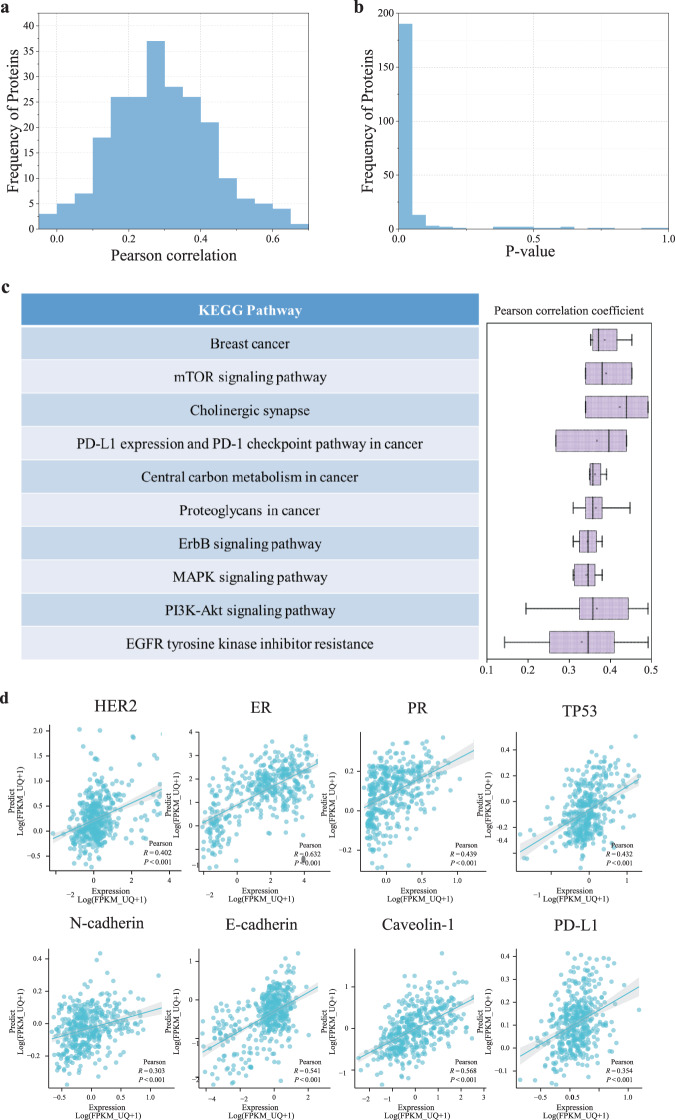
Performance evaluation on the prediction of protein levels of tumor biomarkers. **a**, **b** Showed the histogram of the Pearson correlation coefficients between predicted values and true values of 223 clinically relevant proteins, and the histogram of *p*-values. **c** Showed the KEGG pathways the one-third of well-predicted proteins enriched in (left), and the boxplots of the Pearson coefficients of proteins involved in each signal pathway (right). Boxplot box edges and center line indicate quartiles (25th, 50th, and 75th percentile) of Pearson correlation coefficients, the whiskers represent the range of the data. **d** Scatter plots of the proteins closely associated with targeted therapy and immunotherapy in breast cancer.

We need to make it clear that we used multi-task learning to simultaneously predict the expression level of 223 biomarker proteins. This is quite different from prior methods that trained a predictive model per gene or genetic mutant^15,16,28^. For the evaluation of this regression task, we used the Pearson correlation coefficient (*r*) between the RPPA-measured values and predicted values as the evaluation metric. Similar to previous studies^19^, we considered a prediction to be significantly different from the random baseline value if the *p*-value associated with its coefficient was below 0.05, after applying Benjamini–Hochberg (BH) correction to account for multiple-hypothesis testing. On the TCGA-BRCA cohort, almost all proteins yield significant prediction results. As shown in Fig. 2a, b, 220 out of 223 proteins (99%) were predicted with a statistically significant correlation under BH correction. The average correlation coefficient reached 0.292, of which 164 proteins obtained a correlation coefficient >0.2 and 16 proteins >0.5. We have also tried to validate the model generalizability on protein level prediction but failed to find any other cohort with matched RPPA data and pathology images. As a result, we evaluated our model on the TCGA-BRCA cohort by 5-fold cross-validation. The results showed that our model achieved consistent performance across each fold (see Supplementary Fig. 1), which we believe provides evidence of our model’s robust generalizability.

Among the tumor biomarkers, we paid close attention to the proteins that are clinically relevant to breast cancer, such as estrogen receptor (ER), progesterone receptor (PR), and HER2. Expectedly, our model showed strong predictive power on these biomarkers, as shown in Fig. 2(d). For the estrogen receptor ER-*α*, positive in 70% of breast cancer and a key biomarker for breast cancer diagnosis^29^, our method got *r* = 0.632 correlation coefficient. Progesterone receptor (*r* = 0.439) is induced by ER-*α* and plays an important role in regulating ER-*α* protein, thereby it functions as an important biomarker for breast cancer treatment and prognosis^30^. Highly expressed PR in luminal A type breast cancer indicates a good prognosis^31^. HER2 (*r* = 0.402) overexpression is present in 20–30% of breast cancer and is associated with higher malignancy^32^. The tumor suppressor factor TP53 (*r* = 0.432) has mutated in 80% of triple-negative breast cancers^33^. Caveolin-1 (*r* = 0.568) high expression is a biomarker of more malignant tumors and poor prognosis^34^. PD-L1 (*r* = 0.354) is an immune-related biomarker expressed on the surface of several cell types. Its high expression means potential response to immunotherapy, specifically PD-1/PD-L1 immune checkpoint inhibitors^35^. E-cadherin (*r* = 0.541) and N-cadherin (*r* = 0.303) are epithelial-mesenchymal transitions (EMT)-related biomarkers that confer higher invasion, metastasis, and drug resistance^36^. Moreover, we chose one-third of well-predicted proteins (*n* = 75) to run functional enrichment analysis, and the result was shown in Fig. 2c, from which we found that the well-predicted proteins are enriched in the oncogenic, drug-resistance, and tumor-driven signaling pathways.

### Self-supervised contrastive learning significantly improved predictive performance

**Fig. 3:**
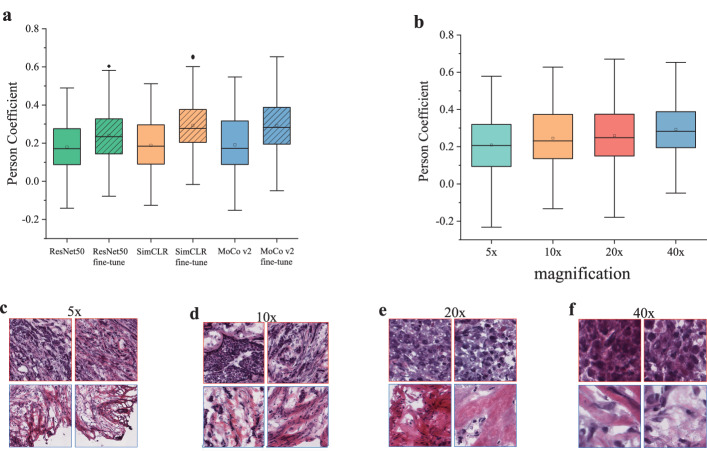
Performance comparison among different contrastive learning methods and multiple image magnification. **a** Boxplots of the Pearson correlation coefficients between the real and predicted protein levels using the tile features extracted by ResNet50, SimCLR, MoCo v2, as well as their fine-tuned versions, respectively. **b** Boxplots of the Pearson correlation coefficients were acquired using the tiles at magnification of ×5, ×10, ×20, and ×40. Boxplot box edges and center line indicate quartiles (25th, 50th, and 75th percentile) of Pearson correlation coefficients, the whiskers represent the range of the data, with outliers (defined as values beyond 1.5 times the interquartile range) plotted as a black rhombus. **c–f** Show some representative tiles at magnification of ×5, ×10, ×20, ×40.

To verify that the pre-trained encoder by contrastive learning extracted histologic features generalized to various downstream tasks, we compared two popular contrastive learning models (SimCLR and MoCo v2) to the baseline ResNet50 network. The ResNet50 was trained on ImageNet and transferred to the prediction task of protein levels. The contrastive learning used the ResNet50 as a backbone network. When the contrastive learning-based pretraining finished, the ResNet50 encoder was frozen in the transfer learning for the protein levels prediction task. For a comprehensive comparison, we also considered fine-tuning the pre-trained ResNet50 encoder by appending a fully connected layer to predict the protein levels. As shown in Fig. 3a, all the models pretrained by contrastive learning outperformed the baseline ResNet50 model. Also, we found that fine-tuned models achieved better predictive power than their respective frozen ones. The experimental result demonstrated that contrastive learning effectively captures expressive and informative features that can be transferred to improve the performance of downstream tasks.

We were also interested in the effect of different resolutions of the scanned pathology images on the predictive performance. We have tested the ×40, ×20, ×10, and ×5 magnification, and the experimental results show that higher magnification achieved better performance in the prediction of protein levels. Figure 3b shows the boxplots of correlation coefficients at different resolutions, the ×40, ×20, ×10, and ×5 magnification yield 0.292, 0.259, 0.246, and 0.209, respectively. For intuitive proof, we visually checked a few exemplar tiles from different resolution slides, as shown in Fig. 3c–f, and found that higher resolution slides convey more detailed information about the morphological structure and histopathological feature. We speculated that higher magnification enables the model to better capture the molecular phenotypic feature of pathological tissues.

### Prognostic scores based on computational pathological features effectively stratify patients

**Fig. 4:**
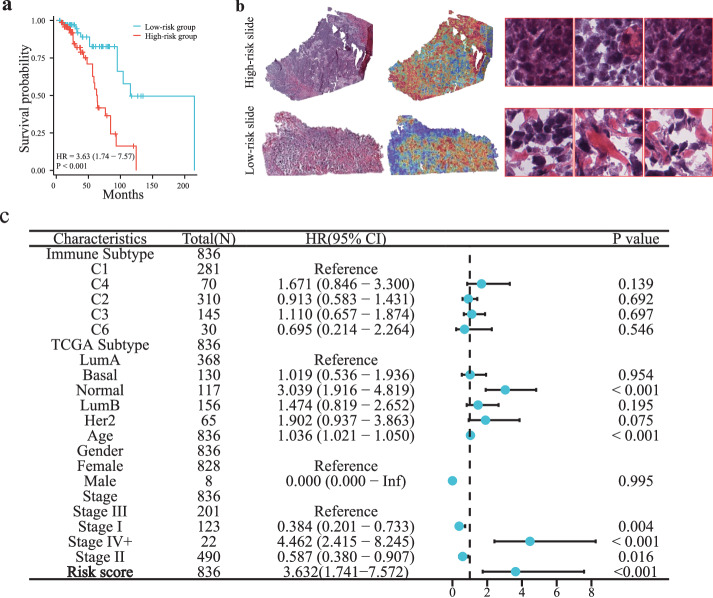
Assessment of the established prognostic risk model based on computational histopathology feature. **a** Survival analysis for the stratified breast cancer patients of TCGA-BRCA cohorts using the risk scores based on computational pathological features. **b** Shows two representative pathology images with high-risk score and low-risk score and their corresponding attention heatmaps, as well as some exemplar tiles. **c** The forest plots of univariate Cox regression based on the computational histopathology feature and clinical variables on the TCGA-BRCA cohort. The forest plot showed the hazard ratios and confidence intervals of each factor, including the prognostic score, age, gender, TCGA subtypes, immune subtypes, and clinical stages.

After aggregation of tile-level features by attentive pooling strategy, we obtained the latent representation of a whole slide image. Following the feature aggregation, we further tested whether the computational pathological feature is indicative of the prognosis of breast cancer patients. For this purpose, a Cox proportional hazards regression model was developed to estimate the survival risk (see the section “Methods”). According to the established prognostic risk score, the breast cancer patients (*n* = 836) of the TCGA-BRCA cohort were stratified to the high-risk group and the low-risk group, and the survival analysis curve was shown in Fig. 4a. The result showed that the 5-year survival time of the high-risk group was significantly lower than that of the low-risk group (HR = 3.63, *p*-value < 0.05), and the concordance index (C-index) of the survival model was 0.64. Moreover, we compared the histopathological difference between the high-risk and low-risk pathology images. As shown in Fig. 4b, it can be found that the high-risk slide contains more tumor regions (high attention score regions in the heatmap) and conveys more invasive histologic features, compared to the low-risk slide. These results showed that our method automatically captured histologic features that were indicative of patient prognosis, which was different from other methods based on manually annotated ROIs. We performed univariate Cox regression on the computational histopathology feature and clinical variables, including tumor staging, molecular subtype, gender, and age, and calculated their hazard ratios on prognosis. Figure 4c demonstrated that the risk score of the pathological feature derived by our model was significantly superior to the values of other clinical variables.

### Computational pathology predicted trastuzumab treatment response

We went further to explore the capacity of computational pathology in predicting the drug response of breast cancer. The Yale trastuzumab response cohort^37^ allows us to run this exploratory experiment. This cohort included the patients with a pre-treatment breast core biopsy with HER2 positive invasive breast carcinoma who then received neoadjuvant targeted therapy with trastuzumab ± pertuzumab prior to definitive surgery. A case was designated as responder if the pathological examination of surgical resection specimens did not report residual invasive, lympho-vascular invasion or metastatic carcinoma, and otherwise non-responders. After removal of the H&E images without enough detectable foreground tissue by redefined area threshold, we obtained 75 cases (34 responders and 41 non-responders) from the Yale trastuzumab response cohort.

**Fig. 5:**
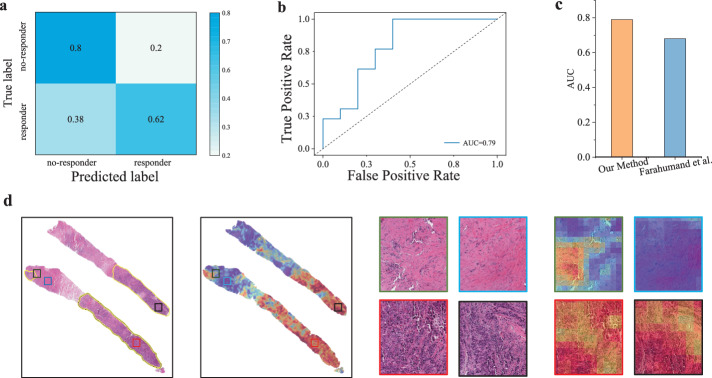
Prediction performance of drug response on Yale trastuzumab response cohort. **a** and **b** showed the confusion matrix and ROC curve of the predicted drug response on the Yale trastuzumab response cohort. **c** Shows the AUC values achieved by our method and Farahmand et al.^37^ (**d**) showed two pathology images with labeled tumor regions by a Yale pathologist and the heatmaps generated by our model (left), as well as some tiles randomly selected from the manually annotated regions (right). The tiles with black and red borders represent the tumor area, blue represents the normal area, and green represents the junction area.

As the HER2 status is used as the clinical biomarker for trastuzumab response to breast cancer, we used only the RPPA-level of HER2, rather than the clinical treatment outcomes, to fine-tune the model pretrained by contrastive learning. Thereafter, the fine-tuned model was directly used to predict the trastuzumab treatment response. This would empower our method to approach clinical practice and output actionable suggestions. As shown in Fig. 5a–c, our method achieved a 0.79 AUC value for the prediction of responders and non-responders, which is much better than the 0.68 obtained by the previous study^37^. Of note, our model was trained by unannotated slides but achieved comparable performance to the previous model trained using pathologist-annotated slides with invasive tumor cells area. Again, we visualized a heatmap generated by the learned attention scores and compared the highly scored regions to the tumor regions annotated by the pathologists of Yale University. Figure 5d demonstrated high consistency between them, and the tiles randomly picked out from high- and low-scored regions agree remarkably with the regions marked as normal and tumor. These results supported the feasibility of image-based biomarkers to predict trastuzumab treatment and the ability of the deep learning model to recognize morphological changes related to treatment results.

### Spatial transcriptomics validated spatial localization of tumor biomarkers

**Fig. 6:**
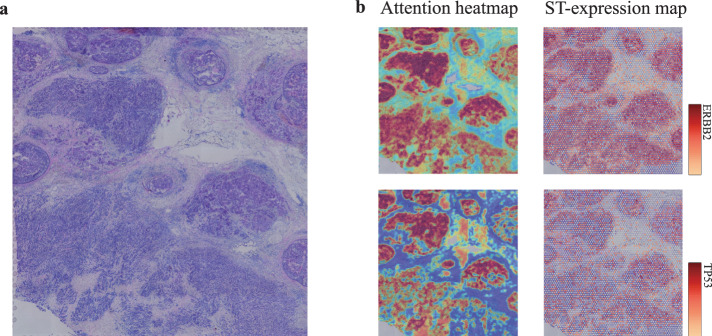
Spatial visualization of spatial transcriptomics and attention score-derived heatmaps of two biomarker genes. **a** Original pathological section used for spatial transcriptome sequencing. **b** Heatmaps showing the ERBB2 and TP53 spatially expressed landscapes generated by our model and spatial transcriptomic data.

Spatial transcriptomics measures the RNA abundance at a high spatial resolution enable us to evaluate our model capacity in the spatial localization of tumor biomarkers. For this purpose, we used the RPPA-measured protein levels of two typical biomarkers, HER2 and TP53, to fine-tune our model. Next, we generated the heatmaps using the learned tile-level attention scores. Correspondingly, we visualized the spatial expression of HER2 and TP53 genes. As shown in Fig. 6, these heatmaps showed that the highly-scored regions were notably consistent with the high expression region of the spatial transcription for both HER2 and TP53. This finding showed that our method effectively performed spatial deconvolution of tumor biomarkers based on conventional H&E staining sections.

## Discussion

Proteomics plays a crucial role in cancer research and translational medicine by identifying key biomarkers for treatment and prognosis. Variations in protein expression levels are indicative of alterations in gene expression, which correspond to changes in cell and tissue phenotypes. By examining these variations, researchers can gain insights into the underlying molecular mechanisms of cancer and develop more effective diagnostic and treatment strategies. Proteomics also has the potential to improve the accuracy of personalized medicine by providing more comprehensive information about an individual unique molecular profile. With the rapid advancement of computational pathology, it has been found that histopathological features are predictive of gene mutations and microsatellite instability. However, few studies have focused on inferring protein levels from pathology images.

While comprehensive molecular tests, such as immunohistochemistry, are difficult to scale to hundreds of biomarkers, tissue sections stained with hematoxylin and eosin are ubiquitously available. Therefore, we set about to predict the protein levels of tumor biomarkers via computational pathology. We hypothesized that these routine tissue sections contain information about established and candidate biomarkers so that molecular biomarkers could be inferred directly from digitized whole-slide images (WSIs). The rationale for this hypothesis is that protein level changes in cells cause changes in cellular function, which influence cell morphology. The underlying molecular profiles eventually dominate the histological characteristics, resulting in higher-order genotype-phenotype correlations. In fact, our experiments have demonstrated that pathological features faithfully reflected the protein levels in the specimen. In our practice, we collaborated with two pathologists in an attempt to discover visual morphological features related to specific protein expression, such as HER2 protein (see Supplementary Fig. 2 for representative tiles). Although our analysis has not yielded significant results supporting the correlation between the morphological features and protein expression levels, we believe that deep learning can extract features, which are higher-order and beyond human visual capabilities, closely related to specific protein expression levels.

There is increasing evidence that solid tumors exhibit significant tumor heterogeneity, namely, each tumor cell has distinct molecular genetic and phenotypic characteristics. This heterogeneity results in differences in growth rate, invasiveness, and drug sensitivity among tumor cells. Targeted drugs can only kill cells expressing specific target proteins, while a small number of drug-resistant tumor cells survive and proliferate, leading to tumor recurrence and progression. Although single-cell sequencing provides high-resolution data to reveal tumor heterogeneity, its high cost and long turnaround time hinder its widespread adoption in routine clinical examinations. In this work, whole slide images were divided into a large number of small tiles, each assigned a weight reflecting the importance of molecular expression levels within different regions of the tumor. This actually provides a fast and inexpensive alternative method for exploring tumor heterogeneity and drug response. Our method demonstrated the potential in predicting the response of breast cancer patients to specific targeted drugs. In predicting the drug response on the Yale trastuzumab response cohort, our image encoder tuned by protein levels data promoted the performance by 11% compared to the original study. Although the current method based on pathological images has not reached clinically applicable standards, we believe that with the accumulation of data, especially the development of spatial transcriptomics, the new computational pathology-based methodology will emerge to provide new insights into tumor heterogeneity from a spatial perspective.

Notwithstanding, our study is not without its shortcomings. First, the quality of our work was impeded by missing data and other related issues. The retrospective cohort we used was plagued with specific information biases and data omissions. For instance, some cases in the TCGA-BRCA cohort lacked clinical data, necessitating the imputation of missing values, which may introduce noise information. Also, due to the paucity of large-scale RPPA data in the external validation cohort, we are unable to ascertain the generalizability of our method in predicting protein levels on external independent datasets. Second, there existed a significant discrepancy between different H&E staining and digitization systems. We conducted color normalization during image preprocessing, but this may have resulted in the loss of information. Third, when establishing the prognostic model and predicting drug response, we did not take into account clinical variables such as clinical staging, which reflects the degree of tumor invasion and metastasis. We believe the inclusion of clinical variables would effectively improve the accuracy of our predictions. Fourth, we did not establish the correlation between protein expression levels measured by fluorescence in situ hybridization or immunohistochemistry (IHC) and those assayed by RPPA. This imposed limitations on the interpretability and clinical translational application of our method. Finally, the number of samples included in our study was relatively small. To enhance the robustness of our model, we need larger and more diverse samples with matched pathology images, protein expression levels, and other clinical variables that are currently unavailable. Despite these limitations, our method still demonstrated the potential in the quantitative estimation of protein expression levels of tumor biomarkers and their clinical applications.

In summary, we proposed a weakly supervised contrastive learning framework to infer protein levels of tumor biomarkers from breast cancer whole-slide images (WSIs). By pre-training the model on large-scale unlabeled breast cancer WSIs, the computational pathological features showed remarkable performance in various downstream tasks. The method performed well in tumor diagnosis and achieved high performance in predicting clinically relevant protein levels. The model interpretability is demonstrated through spatial visualization of WSIs colored by attention scores. Especially, our method also achieved notable accuracy in predicting the response of breast cancer patients to the drug trastuzumab.

## Methods

### Whole slide images

All digital slides of fresh frozen tissue stained with hematoxylin and eosin (H&E) were obtained from TCGA via the Genomic Data Commons Data Portal. From the TCGA-BRCA cohort, we collected 1978 WSIs of 1093 breast cancer patients, comprising 1579 tumor slides and 399 normal slides. The pathology diagnoses provided by the TCGA database were used as ground truth labels for classification tasks. In our practice, we have also experimented with using Formalin-Fixed Paraffin-Embedded (FFPE) slides of the TCGA-BRCA cohort for protein level prediction. However, we found that the performance using FFPE slides was inferior to fresh frozen slides, and thus chose to use fresh frozen slides in our study.

The CPTAC-BRCA cohort, comprising 642 fresh frozen WSIs from 134 breast cancer patients, was used as an external validation cohort.

Another independent cohort came from the Yale trastuzumab response cohort and was used to evaluate the model’s ability in predicting drug response. The Yale cohort contained 75 FFPE WSIs of 75 cases of breast cancer (34 responders and 41 non-responders). A case was designated as responder if the pathological examination of surgical resection specimens did not report residual invasive, lympho-vascular invasion or metastatic carcinoma, and otherwise non-responders. Note that only the slides with a magnification greater than 20* were included in this study. Supplementary Fig. 3 outlined the process of filtering the data in our study. The study was performed in accordance with the ethics standards of the participating institutions and the tenets of the Declaration of Helsinki.

### RPPA dataset

All RPPA data were downloaded from The Cancer Proteome Atlas (TCPA)^38^. The database collects the RPPA-assayed protein levels of samples that mainly came from TCGA (The Cancer Genome Atlas) tumor tissue sample sets. The RPPA data contains more than 200 proteins covering most cancer signaling pathways such as PI3K, MAPK, mTOR, TGF-b, and WNT pathways. In total, there are 893 RPPA samples came from the TCGA-BRCA cohort. After the removal of the sample without matched WSIs, we obtained 860 matched samples that are used to train the protein level prediction model.

### Spatial transcriptomic data

We acquired the spatial transcriptomic data of a breast cancer specimen using the Visium Spatial Gene Expression protocol from the website. To explore the spatial map of gene expression, we utilized the 10x Loupe Browser, a desktop application that offers interactive visualization capabilities for various 10x Genomics solutions. This enabled us to visualize the spatial landscape of specific biomarker gene expression upon the pathology image.

### Weakly-supervised contrastive learning framework

**Fig. 7:**
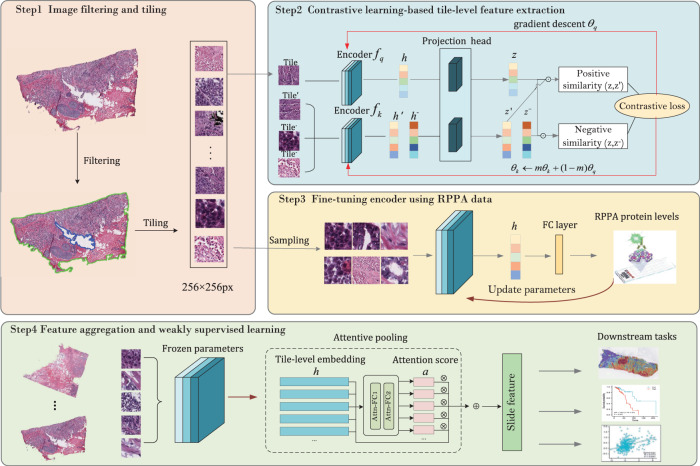
Illustrative flowchart of the proposed wsi2rppa pipeline. The first step preprocessed the WSIs by eliminating regions without sufficient tissue and splitting the slide into tiles. The second step leveraged MoCo v2 contrastive learning to extract tile-level features. The third step tuned the pre-trained encoder using protein levels measured by RPPA assays. Finally, the tile-level features were aggregated using attentive pooling to obtain slide-level features for downstream tasks.

Our learning framework consisted of four steps, as illustrated in Fig. 7. The first step is the preprocessing of whole slide images (WSIs). We eliminated the regions without sufficient pathological tissue and background, and then split each slide into 256*256 px tiles, yielding a total of 17,020,990 tiles with an average of 8706 tiles per slide.

The second stage is the contrastive learning-based feature extraction for unlabeled tiles. Contrastive learning employs self-supervised pretext tasks to learn image embedding and has demonstrated exceptional performance on quite a few tasks. We evaluated two typical contrastive learning algorithms, MoCo v2 and SimCLR, and found that they learned more informative features in comparison to the ResNet50 baseline.

In the third stage, we utilized protein expression levels measured by Reverse Phase Protein Array (RPPA) assays to fine-tune the pre-trained encoder via weakly supervisory signals. Due to the too large number of tiles, we sampled 20% tiles (*m* = 398,426) from 200 randomly selected slides to run the fine-tuning task. Finally, we employed attentive pooling to aggregate tile-level features into slide features ready for downstream tasks, including tumor diagnosis, prediction of biomarker gene expression levels and drug treatment outcome, and establishment of prognostic score. Supplementary Fig. 3 shows the consort diagram describing the detailed number of samples of each cohort, and Supplementary Fig. 4 illustrates the cohorts and features used in downstream tasks.

### Preprocessing of whole slide images

Due to the ultra-high resolution of pathological images, which can reach up to gigapixels, they are not immediately suitable for input into a deep-learning model. As a result, we divided each whole slide image into small squares known as tiles or patches. The Python package OpenSlide was utilized to read a slide into memory, and the Otsu algorithm was then employed to differentiate between tissue and background regions. Following segmentation, the tissue area was divided into 256*256 px tiles.

### Contrastive learning for feature extraction

Given a large scale of unlabeled tiles, we leveraged the contrastive learning-based pretraining to learn an encoder to produce latent representations for the tiles. In this study, we used MoCo v2 for the pretraining. We also tested another contrastive learning method, SimCLR, and found that MoCo v2 achieved better performance in downstream tasks.

Formally, contrastive learning learns to extract features by minimizing the distance between the representations of positive pairs (e.g. image and its augmentation) and maximizing the distance between representations of negative pairs (e.g. different images). Suppose a tile *x* has its augmentation ${x}^{{\prime} }$. We use a CNN network as the backbone encoder *f* to obtain their embedding *h* and ${h}^{{\prime} }$, where *h* = *f*(*x*). Subsequently, *h* goes through a projection head *g* composed of two fully connected layers to produce vector *z* and ${z}^{{\prime} }$, where *z* = *g*(*h*). The contrastive loss in a minibatch was defined as follows:

1 $$L=-{\rm {log}}\frac{\exp ({\rm {sim}}({z}_{i},{z}_{i}^{{\prime} }))}{\mathop{\sum }\nolimits_{k = 1}^{K}\exp ({\rm {sim}}\left({z}_{i},{z}_{k}\right))}$$

in which sim() is the similarity function, and *K* is the number of negative samples cached in memory bank. We adopted a simple similarity defined as below:

2 $${\rm {sim}}\left({z}_{i},{z}_{j}\right)=\frac{{z}_{i}^{{\rm {T}}}{z}_{j}}{\tau | {z}_{i}| | {z}_{j}| }$$

where *τ* is an adjustable temperature parameter. Once the pretraining was finished, we discarded the projection head and used the trained encoder to obtain the feature of tiles.

We used ResNet50^39^ as the backbone network and loaded the pretrained weights on ImageNet. The SGD optimizer was used, the learning rate of the backbone network was set to 0.03, the weight decay rate was 0.0001, and the momentum was 0.9.

### Feature aggregation

For downstream tasks, we need to aggregate individual tile-level features into slide-level features. We employ gated-attention pooling^40^. Suppose a slide has *N* tiles S={*p*_1_, *p*_2_, . . . *p*_*N*_}, we got the feature *h*_*i*_ using the pretrained encoder for tile *p*_*i*_, the gated-attention pooling is essentially the instance-level weighted average pooling:

3 $$S=\mathop{\sum }\limits_{i=1}^{N}{a}_{i}{h}_{i}$$

and

4 $${a}_{i}=\frac{{\rm {exp}}({w}^{{\rm {T}}}(\tanh (V{h}_{i})\cdot {\rm {sigm}}(U{h}_{i})))}{\mathop{\sum }\nolimits_{j = 1}^{N}{\rm {exp}}({w}^{{\rm {T}}}(\tanh (V{h}_{j})\cdot {\rm {sigm}}(U{h}_{j})))}$$

where *U* and *V* are trainable parameter matrices, ⋅ denotes element-wise multiplication. The introduction of learnable parameters that are updated iteratively during the training process makes the model highly flexible and adaptable to various downstream tasks. Furthermore, after training, the attention weights reflect the significance of tile-level features for the downstream task, rendering the model interpretable.

### Tumor diagnosis

We formulated the tumor diagnosis as the binary classification task. The ground truth labels (tumor or normal) came from the TCGA-BRCA cohort. We employed a fully connected layer plus a softmax layer as the prediction model that took the slide feature as input. The cross-entropy was used as the loss function for the classification task.

### Protein level prediction

The protein level estimation for 223 tumor biomarkers was formulated as a multi-task regression task. Taking as input the slide features, we adopted a multi-task learning model with a fully connected layer and an output layer. The output layer had 223 nodes and each of them correspond to the level of a protein. In our practice, we have also tested multiple hidden layers architecture and found that a single hidden layer could achieve superior performance in the regression task. The mean squared error (MSE) was used as a loss function:

5 $$L=\frac{1}{N}\mathop{\sum }\limits_{i=1}^{N}{({y}_{i}-{y}_{i}^{{\prime} })}^{2}$$

where *y*_*i*_ is the measured level of protein *i* by RPPA assays, ${y}_{i}^{{\prime} }$ is the predicted level, and *N* is the total number of proteins.

### Drug response prediction

The Yale trastuzumab response cohort was used to evaluate the model’s capacity to predict drug response. As a case was designated as responder or non-responder, we formulated the prediction of drug response from pathological features as binary classification problems and the cross-entropy loss function was used.

### Prognosis model

We used slide-level features to predict prognostic risk for each breast cancer patient. Given the slide feature *x*, time period *T*, and event indicator *E*, we used a Cox proportional hazard regression model based on deep learning to estimate prognostic risk. The hazard function is defined as:

6 $$h(t| x)=\mathop{\lim }\limits_{\bigtriangleup t\to 0}\frac{{\rm {Pr}}(t\le O\le t+\bigtriangleup t| O\ge t;x)}{\bigtriangleup t}$$

s It estimates the instantaneous death rate of individual *x* at time *t*. The Cox proportional hazard regression models it as

7 $$h(t| x)={h}_{0}\left(t\right)\exp ({\theta }^{{\rm {T}}}x)$$

where *h*_0_ represents the baseline risk function at time *t*, which is estimated by the Breslow estimation method. Our prognostic model was constructed and trained using the Pycox Python package. During training, the follow-up status and survival time are used to compute censored data and risk sets. To estimate the parameters *θ*, we minimized the negative log partial likelihood:

8 $$L(\theta )=-\mathop{\sum}\limits_{i:{E}_{i}=1}\left({\theta }^{{\rm {T}}}{x}_{i}-{\rm {log}}\mathop{\sum}\limits_{j\in R({t}_{i})}{\rm {exp}}({\theta }^{{\rm {T}}}{x}_{j})\right)$$

*R*(*t*_*i*_) is the risk set of time *t*_*i*_, which represents the set of patients who may be at risk at time *t*, and *E*_*i*_ = 1 indicates the death event.

### Reporting summary

Further information on research design is available in the Nature Research Reporting Summary linked to this article.

### Supplementary information

Supplementary file

Reporting Summary

## Data Availability

The whole slide images and corresponding labels of the TCGA-BRCA cohort from the TCGA database are available at https://portal.gdc.cancer.gov/. The CPTAC-BRCA whole-slide with corresponding labels and the matched RNA-seq data are available from the NIH cancer imaging archive (https://cancerimagingarchive.net/datascope/cptac). All RPPA data from The Cancer Proteome Atlas (TCPA) database is available at https://www.tcpaportal.org/tcpa/. The whole slide images and drug response data from the Yale trastuzumab response cohort are available at the TCIA database https://wiki.cancerimagingarchive.net/. The spatial transcriptomic data of a breast cancer specimen from 10x genomics are available at https://www.10xgenomics.com/. All other data supporting the findings of this study are available from the corresponding author upon reasonable request. Source data are provided in this paper.

## Code availability

All code was implemented using PyTorch as the primary deep-learning library. The complete pipeline for processing WSIs as well as training and evaluating our model are available at https://github.com/hliulab/wsi2rppa.
