# Supplementary material for: Deep learning infers clinically relevant protein levels and drug response in breast cancer from unannotated pathology images
Source: NPJ Breast Cancer. 2024 Feb 27;10:18. doi: 10.1038/s41523-024-00620-y (PMC10899601; doi:10.1038/s41523-024-00620-y)
Supplement: Supplementary file 1 — Supplementary file [file 41523_2024_620_MOESM1_ESM.pdf]

Supplementary file of wsi2rppa

Hui Liu, Xiaodong Xie and Bin Wang*

Five-fold cross validation for protein level prediction

We have attempted to validate the model generalizability for protein level prediction on external validation cohort. Despite our best efforts, we failed to find any other cohort with matched RPPA data and pathology images. So, we evaluated our model on the TCGA-BRCA cohort by 5-fold cross-validation. The results showed that our model achieved consistent performance across each fold (see Figure S3). We think the results support our model's robust generalizability.

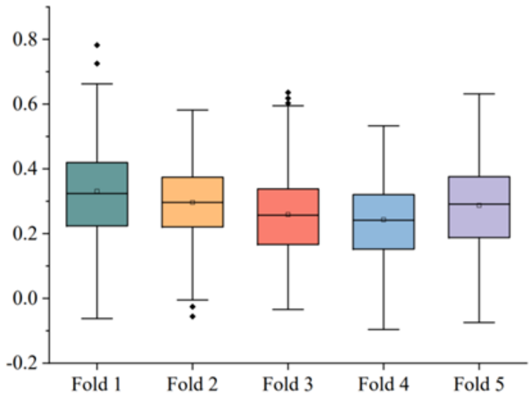

Supplementary Figure 1 The boxplots of the Pearson correlation coefficients between prediction and real protein levels on TCGA-BRCA cohort by 5-fold cross-validation. Boxplot box edges and center line indicate quartiles (25th, 50th, and 75th percentile) of Pearson correlation coefficients, the whiskers represent the range of the data, with outliers (defined as values beyond 1.5 times the interquartile range) plotted as black rhombus.

Visual morphological features for protein expressions

We collaborated with two pathologists in an attempt to discover visual morphological features related to protein expression. Specifically, we used the HER2 protein as an example. After training the model using HER2 expression data, we employed attention scores to identify high-scored and low-scored tiles (40x). The tiles that did not contain tumor cells were filtered out, and only one tile from the same slide was retained. In this manner, we obtained 200 tiles for each groups with high and low HER2 expression (see Figure 2 for representative tiles). Next, two experienced pathologists (Dr Meihua Wang from Changzhou Cancer Hospital in Jiangsu, China. Dr. Dachuan Zhang from the Third Affiliated Hospital of Soochow University, Changzhou, Jiangsu, China.) were asked to independently review these tiles and evaluated visually interpretable morphological features, including tumor cell shape, nuclear size, nucleolar staining, proportion of gland formation, and mitotic count. Based on these morphological feature, we attempted to correlate these features with the expression of HER2. Regrettably, we did not obtain statistically significant results. We further tested several other proteins, including TP53 and ERBB2, but did not obtain meaningful results.

Although our exploration did not yield significant evidence to support the correlation between pathological morphological features and protein expression levels, we believe that deep learning can extract higher-order features, which are high-dimensional and beyond human visual capabilities, closely related to specific protein expression levels. Furthermore, the heat map generated by the attention score display the regions corresponding to high-level protein expression, laying the foundation for us to further explore visually interpretable features. In future work, we will continue to explore this issue and endeavor to uncover additional morphological features that can quantitatively explain protein expression.

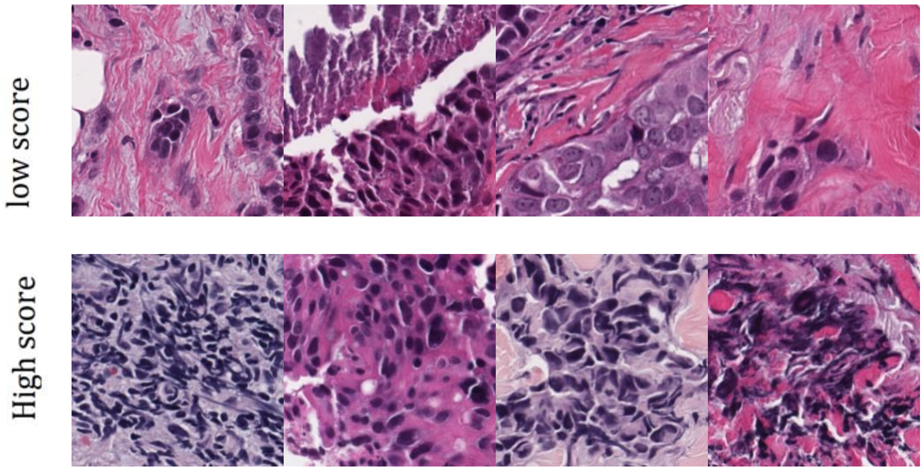

Supplementary Figure 2 Representative tiles with high and low attention scores corresponding to the expression level of HER2

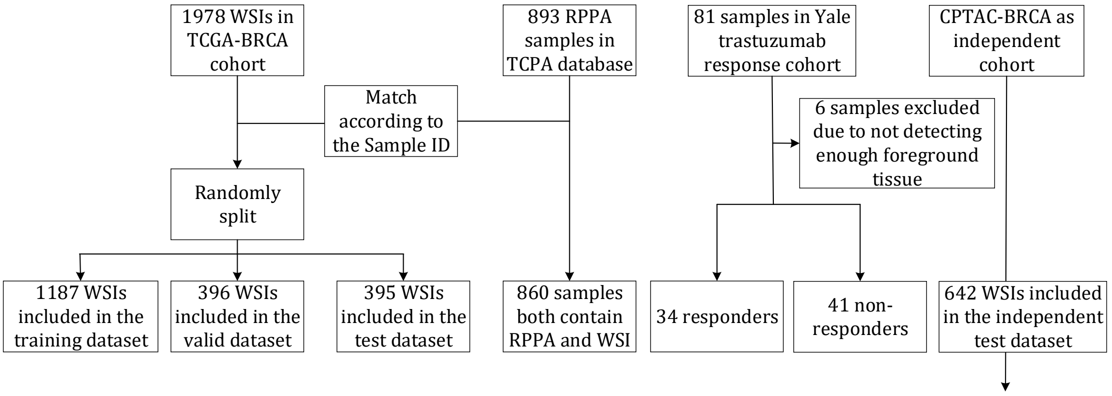

Supplementary Figure 3 Descriptive diagram of the patient cohorts used in our study

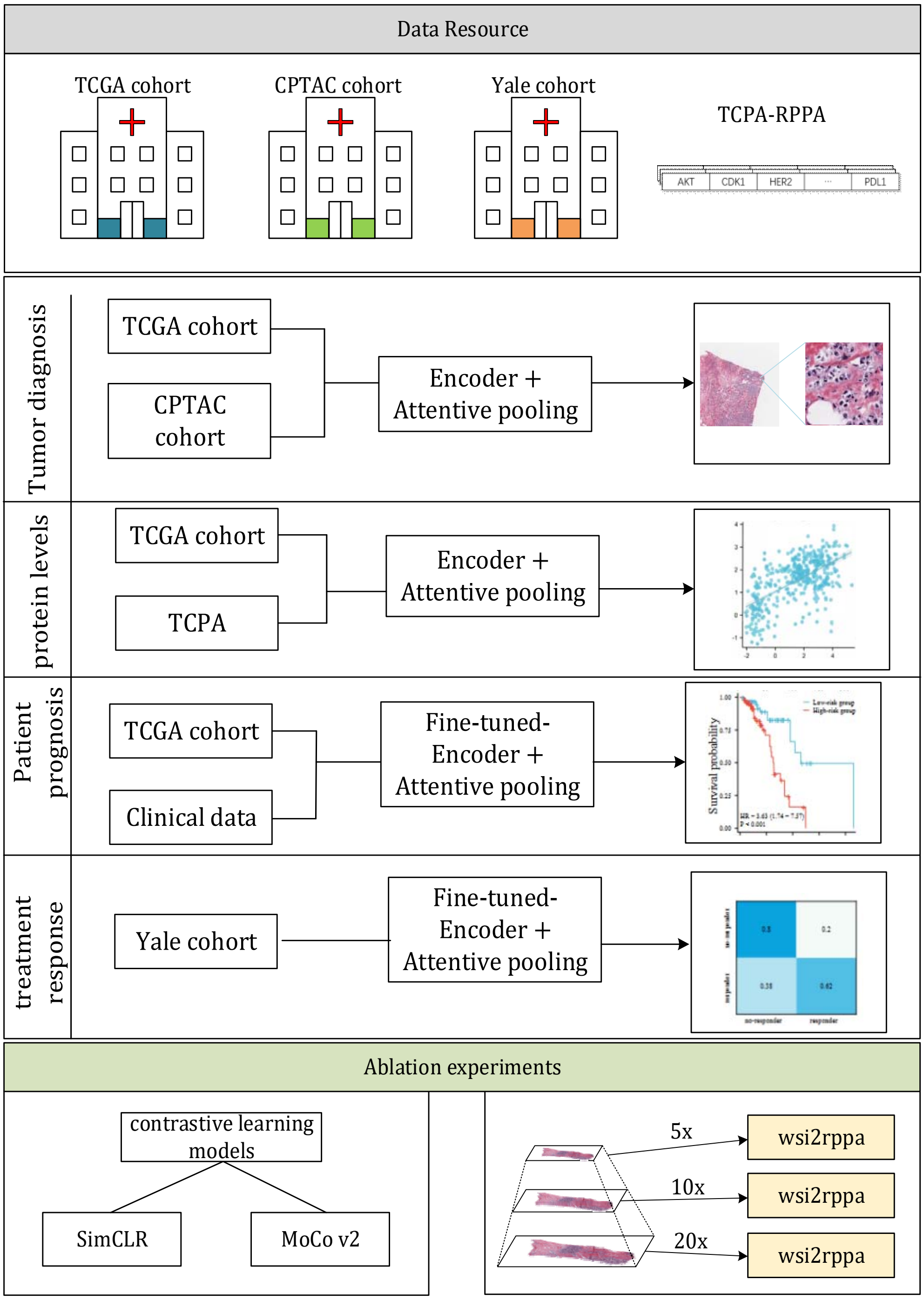

Supplementary Figure 4 Diagram presents the cohorts and features used in downstream tasks
