# Supplementary material for: Deep learning infers clinically relevant protein levels and drug response in breast cancer from unannotated pathology images
Source: NPJ Breast Cancer. 2024 Feb 27;10:18. doi: 10.1038/s41523-024-00620-y (PMC10899601; doi:10.1038/s41523-024-00620-y)
Supplement: Supplementary file 2 — Reporting Summary [file 41523_2024_620_MOESM2_ESM.pdf]

nature portfolio

Corresponding author(s): Bin Wang

Last updated by author(s): Dec 24, 2023

Reporting Summary

Nature Portfolio wishes to improve the reproducibility of the work that we publish. This form provides structure for consistency and transparency in reporting. For further information on Nature Portfolio policies, see our Editorial Policies and the Editorial Policy Checklist.

Statistics

For all statistical analyses, confirm that the following items are present in the figure legend, table legend, main text, or Methods section.

n/a	Confirmed	
☐	☒	The exact sample size (*n*) for each experimental group/condition, given as a discrete number and unit of measurement
☐	☒	A statement on whether measurements were taken from distinct samples or whether the same sample was measured repeatedly
☐	☒	The statistical test(s) used AND whether they are one- or two-sided<br>*Only common tests should be described solely by name; describe more complex techniques in the Methods section.*
☐	☒	A description of all covariates tested
☐	☒	A description of any assumptions or corrections, such as tests of normality and adjustment for multiple comparisons
☐	☒	A full description of the statistical parameters including central tendency (e.g. means) or other basic estimates (e.g. regression coefficient) AND variation (e.g. standard deviation) or associated estimates of uncertainty (e.g. confidence intervals)
☐	☒	For null hypothesis testing, the test statistic (e.g. *F*, *t*, *r*) with confidence intervals, effect sizes, degrees of freedom and *P* value noted<br>*Give P values as exact values whenever suitable.*
☐	☒	For Bayesian analysis, information on the choice of priors and Markov chain Monte Carlo settings
☐	☒	For hierarchical and complex designs, identification of the appropriate level for tests and full reporting of outcomes
☐	☒	Estimates of effect sizes (e.g. Cohen's *d*, Pearson's *r*), indicating how they were calculated

Our web collection on statistics for biologists contains articles on many of the points above.

Software and code

Policy information about availability of computer code

Data collection	The software was developed using Python programming language (Version 3.8). The deep learning model was implemented using PyTorch v1.11. The complete pipeline for processing WSIs as well as training and evaluating our model is available at https://github.com/hliulab/wsi2rppa.
Data analysis	https://github.com/hliulab/wsi2rppa.

For manuscripts utilizing custom algorithms or software that are central to the research but not yet described in published literature, software must be made available to editors and reviewers. We strongly encourage code deposition in a community repository (e.g. GitHub). See the Nature Portfolio guidelines for submitting code & software for further information.

Data

Policy information about availability of data

All manuscripts must include a data availability statement. This statement should provide the following information, where applicable:

- Accession codes, unique identifiers, or web links for publicly available datasets
- A description of any restrictions on data availability
- For clinical datasets or third party data, please ensure that the statement adheres to our policy

The whole slide images and corresponding labels of the TCGA-BRCA cohort from the TCGA database are available at https://portal.gdc.cancer.gov/. The CPTAC-BRCA whole-slide with corresponding labels, and the matched RNA-seq data are available from the NIH cancer imaging archive https://cancerimagingarchive.net/

datascope/cptac. All RPPA data from The Cancer Proteome Atlas (TCPA) database is available at https://www.tcpaportal.org/tcpa/. The whole slide images and drug response data from Yale trastuzumab response cohort are available at TCIA database https://wiki.cancerimagingarchive.net/. The spatial transcriptomic data of a breast cancer specimen from 10x genomics are available at https://www.10xgenomics.com/. All other data supporting the findings of this study are available from the corresponding author upon reasonable request. Source data are provided with this paper.

Research involving human participants, their data, or biological material

Policy information about studies with human participants or human data. See also policy information about sex, gender (identity/presentation), and sexual orientation and race, ethnicity and racism.

Reporting on sex and gender	All data is publicly available, and all information can be found on the following websites:<br>1) https://portal.gdc.cancer.gov/<br>2) https://cancerimagingarchive.net/datascope/cptac<br>3) https://www.tcpaportal.org/tcpa/<br>4) https://wiki.cancerimagingarchive.net/
Reporting on race, ethnicity, or other socially relevant groupings	All data is publicly available, and all information can be found on the following websites:<br>1) https://portal.gdc.cancer.gov/<br>2) https://cancerimagingarchive.net/datascope/cptac<br>3) https://www.tcpaportal.org/tcpa/<br>4) https://wiki.cancerimagingarchive.net/
Population characteristics	Information on characteristics such as gender, age, breast cancer subtype, and cancer staging can be found in the official guidelines.
Recruitment	All data is publicly available. The recruitment process for study participants can be found in the official guidelines.
Ethics oversight	This study employs publicly accessible data in compliance with relevant ethical guidelines.

Note that full information on the approval of the study protocol must also be provided in the manuscript.

Field-specific reporting

Please select the one below that is the best fit for your research. If you are not sure, read the appropriate sections before making your selection.

☒ Life sciences ☐ Behavioural & social sciences ☐ Ecological, evolutionary & environmental sciences

For a reference copy of the document with all sections, see nature.com/documents/nr-reporting-summary-flat.pdf

Life sciences study design

All studies must disclose on these points even when the disclosure is negative.

Sample size	All digital slides of fresh frozen tissue stained with hematoxylin and eosin (H&E) were obtained from TCGA via the Genomic Data Commons Data Portal. From the TCGA-BRCA project, we collected 1,978 WSIs of 1,093 breast cancer patients, comprising 1,579 tumor slides and 399 normal slides. The CPTAC-BRCA cohort, comprising 642 fresh frozen WSIs from 134 breast cancer patients, was used as an external validation cohort. Another independent cohort came from Yale trastuzumab response cohort was used to evaluate model ability in predicting drug response. The Yale cohort contained 75 samples.
Data exclusions	Figure S2 provides an overview of our data filtering process.
Replication	We have conducted multiple tests to ensure the reproducibility of the experiments.
Randomization	The TCGA cohort were randomly split into training, validation and independent test set by 60%, 20% and 20%.
Blinding	Investigators were blinded during data collection.

Reporting for specific materials, systems and methods

We require information from authors about some types of materials, experimental systems and methods used in many studies. Here, indicate whether each material, system or method listed is relevant to your study. If you are not sure if a list item applies to your research, read the appropriate section before selecting a response.

Materials & experimental systems

n/a	Involved in the study
☒	☐ Antibodies
☒	☐ Eukaryotic cell lines
☒	☐ Palaeontology and archaeology
☒	☐ Animals and other organisms
☒	☐ Clinical data
☒	☐ Dual use research of concern
☒	☐ Plants

Methods

n/a	Involved in the study
☒	☐ ChIP-seq
☒	☐ Flow cytometry
☒	☐ MRI-based neuroimaging
